# The Emotional Experience of Pregnancy in a Transgender Man: A Case Report

**DOI:** 10.5935/1518-0557.20260021

**Published:** 2026

**Authors:** Juliana Roberto dos Santos, Caio Parente Barbosa, Bianca Bianco

**Affiliations:** 1 Postgraduation Program in Health Sciences, Centro Universitário FMABC, Santo André/SP, Brazil; 2 Instituto Ideia Fértil, Santo André/SP, Brazil; 3 Discipline of Sexual and Reproductive Health and Population Genetics, Department of Collective Health, Centro Universitário FMABC, Santo André/SP, Brazil

**Keywords:** welcoming, transgender men, identity, transgender individuals, assisted reproduction techniques

## Abstract

The present study aimed to understand the lived experience of a transgender man who underwent pregnancy. The existing literature indicates that many professionals are not prepared to assist transgender individuals, often supporting a heteronormative view of sexuality and reproduction. This case report explored whether the experience of pregnancy impacted the gender identity and experience of masculinity in a transgender man. A clinical case study methodology was employed. Initially, it was hypothesized that pregnancy would neither compromise the participant’s male identity nor weaken his experience as a transgender man. One transgender man expressed a desire to carry his genetic child. Our findings revealed that he began to enjoy the experience and was positively reconnected with his pregnant body by the sixth month of pregnancy. The psychic elaboration of the fantasy that “carrying a pregnancy is not a man’s thing” made him reconsider the feelings of impotence and vulnerability that are traditionally attributed to the females. He had a satisfactory experience with the healthcare team. He actively assumed the role of “father-birthgiver,” demonstrating that fathers are also capable of sensitively responding to a baby’s emotional needs. Although it mobilized complex psychological processes, the pregnancy experience did not cause identity disruption. This study indicates that transgender men can be psychologically and emotionally available for a baby in a state of absolute dependence.

## INTRODUCTION

“Transgender” emerged in the early 1990s as an umbrella term encompassing a broad spectrum of gender identity variations characterized by a divergence between one’s current gender identity and the gender assigned at birth (Costa *et al*., 2018). According to Lanz (2017), transgender expressions are becoming increasingly apparent in the contemporary society. Social advances have enabled transgender individuals of all ages and social classes to openly embrace their identities, thereby “... openly challenging the male-female dichotomy that characterizes the binary gender system prevalent in society”.

However, combating violence is one of the main challenges. According to Transgender Europe (TGEU, 2025), Brazil has had the highest number of transgender murders worldwide for the 15th consecutive year. Despite this devastating reality and difficult experiences in healthcare services, more transgender individuals want to establish families and consider their reproductive futures (Fernández-Basanta *et al*., 2024; Pereira *et al*., 2024).

From the family perspective, the desire to experience pregnancy is not exclusive to women; transgender men with a uterus may also wish to experience gestation.“Some studies have highlighted a lack of preparedness among healthcare teams to assist transgender people, reflecting a heteronormative view of sexuality and reproduction. These professionals require continuous training regarding diversity issues” (Nascimento, 2024; Silva *et al*., 2024).

The present study aimed to understand the lived experience of a transgender man who had undergone pregnancy. This report describes a case to explore whether pregnancy impacted the gender identity of a transgender man. Additionally, we hope that this knowledge will assist multidisciplinary teams, both public and private, in providing the best care to transgender patients seeking assisted reproduction services for family formation, as well as to teams accompanying prenatal care and childbirth.

This study adopted a clinical-qualitative method based on interpretative principles grounded in psychoanalytic listening according to Winnicottian psychoanalysis. This method allows access to the unique psychic meanings produced in the participant’s speech arising from his experience of pregnancy as a transgender man. Listening was not employed for the empirical confirmation of hypotheses, but to capture phenomena such as mutuality ([1969]1994) and primary maternal preoccupation ([1956]2021) described by Winnicott, consistent with the singularity of the participant’s identity journey. The analysis was conducted using a theoretical-clinical articulation between emerging discourses and Winnicottian frameworks, preserving the ethical and non-invasive nature of psychoanalytic listening requirements.

## METHODS

This guideline followed the CARE protocol (EQUATOR Network) and adopted a qualitative approach using the case study method, grounded in the psychoanalytic perspective. Data collection was carried out through a semi-structured interview developed by the researchers to address the study objectives. The format allowed the participant to express himself freely within predefined themes, respecting the spontaneity of the discourse and the subject’s uniqueness.

### Participant

During the search for participants, significant difficulty was encountered in accessing the target population-namely, trans men who had experienced pregnancy. Although many are active on social media, contact attempts through these platforms yielded no responses. Only one trans man agreed to participate in the study, having been referred by a psychologist who knew both the participant and the lead researcher. We also contacted other psychologists, particularly those working in assisted reproduction clinics, but none had treated trans patients who had been pregnant, nor were they aware of any potential participants.

During this recruitment process, we spoke with a trans psychologist who works in psychotherapy with trans patients. She shared the perception that, in her experience, most trans men do not wish to become pregnant. According to her, once they affirm their identity as men, this is the role they inhabit-with no space for experiences typically associated with femininity, such as pregnancy-reflecting a perspective marked by gender binarism.

We consider that the scarcity of responses may be related both to the intense prejudice these individuals face, which may lead to caution regarding exposure, and to potential fatigue, as they are often solicited to participate in research due to the visibility and novelty of the phenomenon.

Thus, this case study consisted of a single participant who, in respect of anonymity, will be referred to as M. He is 32 years old, single, began his gender transition in 2018, and decided to become a father in 2021.

### Instrument

The choice of a semi-structured interview aligns with the psychoanalytic method, which privileges attentive listening and free association as pathways to the unconscious. During the interview, a stance of evenly suspended attention was maintainedin order to identify latent elements, contradictions, and unconscious formations in the participant’s speech.

The semi-structured interview was designed by the researchers and covered the following topics: the participant’s gender transition history and lived experience; the current family structure and the decision to pursue pregnancy, including the conception process; emotions during pregnancy, as well as any doubts or concerns; the birth narrative and the experience of that moment; emotions upon seeing the child for the first time; the initial days with the newborn; and the chosen method of infant feeding. At the end, the participant was asked whether his current family meets the expectations he had prior to becoming a parent.

This approach is based on the proposal of Ocampo and Arzeno (1999), who define the semi-structured interview as a technique that allows the interviewee to construct his own psychological field, enabling him to present his issues in the way he considers most appropriate. Unlike a completely unstructured interview, this method allows the interviewer to make timely interventions to promote the flow of dialogue or explore specific content in more depth.

### Procedures and Ethical Considerations

A one-and-a-half-hour online interview was conducted via Google Meet. The session was audio-recorded and subsequently transcribed. After reading the Informed Consent Form (ICF), the participant agreed to take part in the study, which was approved by the Research Ethics Committee of the ABC School of Medicine.

The signed ICF, initially signed by the researcher, was sent to the participant, who also signed it and returned a copy to the researcher.

### Evidence Analysis

In this qualitative study, methodological triangulation was employed as a strategy to ensure greater robustness and depth in data analysis, as proposed by Minayo (2014). Triangulation allows for the combination of different data sources, collection techniques, or theoretical perspectives, thus fostering a broader and more complex understanding of the studied phenomenon.

The semi-structured interview was audio-recorded (with participant consent) and analyzed not only by the lead researcher but also by two additional psychoanalysts, to broaden the interpretation of the narratives.

This collaborative analysis enabled the confrontation of different perspectives on the empirical material, contributing to the validity of the findings, reducing individual bias, enriching the analysis, and strengthening scientific rigor in qualitative research (Minayo, 2014).

Discourse analysis was conducted, and central analytical categories were defined in line with the study’s objectives: How does this man experience pregnancy? What motivates this man to desire pregnancy, having previously rejected the female gender? And does the experience of pregnancy evoke in him any desire to become a woman?

The material was independently analyzed by the three psychoanalysts using psychoanalytic listening and interpretation, considering both manifest and latent content, as well as possible unconscious formations such as slips, silences, repetitions, and emotions evoked during the interview. At the end of the process, only findings that showed agreement among all three professionals were included in the present study.

## CASE DESCRIPTION

A transgender man, identified as M. and aged 32 years, became pregnant naturally after attempting for a year and a half. Previously, he underwent gender-affirming mastectomy while preserving his uterus and ovaries. Regarding his gender transition history, M. reported that he never felt comfortable being called by his birth name and preferred to be addressed by a gender-neutral nickname. When he met another transgender man who resembled the ideal man that he wished to become, he found a way to exist. The mode M. embraced was through the body, and began his gender transition process.

How could he claim to be a man without showing it on his body? The idea of transforming his body came to M. as what made him a transgender man. Without hormones, he felt *“weak,” “vulnerable,”* and *“impotent,”* as if he *“lacked spinach,”* paraphrasing Popeye the sailor, a classic cartoon character who gained superhuman strength by eating spinach.

When “transcentring” - that is, relating for the first time with a transgender woman - M. experienced a rich and highly significant experience for his being and for demystifying issues that, according to him, were *“patriarchal”* and *“prejudiced.”* In the past, he considered the idea of body modification as establishing a new existence in which the old body would be hidden. By engaging in a romantic relationship with a transgender woman, he could use his body and felt happy to enjoy the advantages his body could offer him.

It was then, as he adapted to a vigorous body, that he considered the idea of becoming a father and having a genetic child: *“She (the trans woman) said I could gestate even being a man. I thought it was crazy, but then I liked the idea-I could gestate, have a genetic child, and still be a man.”*

Later, M. entered into a relationship with another transgender woman and they decided to build a family together.

## DICUSSION AND RESULTS

With a new perception of his body, M. was willing to carry a child *“of his own.”* Having a child *“of his own”* relates to the idea of ongoing life. However, this implies that consanguineous bonds guarantee the emergence of affection in intersubjective relationships.

This intense desire led M. to consider pregnancy. At first, he thought it would be simple to carry the pregnancy for nine months: *“I thought about hiding my belly with loose clothes.”* He accepted using his body and the benefits of his reproductive system but did not imagine himself as an openly pregnant man.

Upon deciding to pursue pregnancy, M. consulted his endocrinologist, who recommended suspending the hormones used for bodily modifications, allowing ovulation to resume and enable pregnancy. The one-and-a-half years spent trying to conceive without success was emotionally challenging, leading him to contemplate quitting. During this period, sexual relations focused exclusively on conception.

M. discovered his pregnancy at three months. His belly was discreet and he was adapting to his pregnant body and the new reality. By the sixth month, M. had begun to connect deeply with his baby. *“I loved feeling my son inside my belly, feeling him move, I was anxious to see his little face...”* He already loved him, prepared the layette, and did a maternity photo shoot, while affirming the value of his body. He began to enjoy the pregnancy and appreciate the bodily changes: *“I felt beautiful.”* M. came to terms with the body he was born with, a female body.

Throughout the pregnancy, M. was supported by a doula who offered significant assistance. She said, *“Your strength is in the uterus; if it weren’t for the uterus, none of us would be here...”* This statement helped him rewrite his story and re-signify his body as a territory of creation. This transition became more flexible and awakened the desire for natural childbirth. The father who gestated allowed his body to function for the child’s birth; he did not just use the uterus but integrated the whole body. This experience also demonstrates that people with a uterus, regardless of their gender identity, may desire to gestate and experience parenthood.

During prenatal care, M. was followed by an obstetrician in the São Paulo state public health system (SUS): *“I was very well attended throughout my prenatal care, and on the day of delivery, only women professionals were with me...”*

Before this process, M. considered being a woman as being powerless. He attributed masculine and feminine meanings to non-vulnerability and vulnerability, respectively. The elaboration of the fantasy that he couldn’t pregnancy because was a *“woman’s thing”* made him reconsider his fantasy of impotence and vulnerability. Traversing this elaboration allowed him to connect with the baby. *“I am a transgender man who has something feminine.”* He was able to integrate the masculine and normalize the feminine energy.

The contact with the newborn baby moved him deeply. M. was touched and apprehensive before holding the fragile and delicate human being. M.’s delivery was assisted by the mother. Right after birth, he offered the baby to the mother, saying: *“Here he is, he’s yours too!”*

However, in his narrative of caring for and welcoming his child, he assumed the role of father-birthgiver rather than mother*: “She never stayed alone with the baby, he cried a lot and she couldn’t calm him down, I left work to care for my son.”* This is an inseparable combination, the baby is not alone. The presence of a caring human prevents the baby from collapsing physically or psychically.

The English pediatric psychoanalyst Winnicott (2021), states only a sensitized mother can put herself in the baby’s place and meet his needs, which initially are bodily but gradually transform into “ego needs.” He further states that an adoptive mother or a woman available to care must possess the ability to “become ill” in the sense of reaching the stage of primary maternal preoccupation and identifying with the baby and fully adapting to him. Failures of the mother are perceived by the baby as “threats to personal existence” (p. 497).

However, it is not only the mother who can become sensitized and put herself in the baby’s place to respond to his needs. Our work reveals that transgender men also have the capacity to “become ill” (i.e., to develop primary maternal preoccupation).

Nonetheless, although a uterine model emerged in M., which facilitated caregiving and increased his availability for this task, pregnancy itself does not thoroughly complete this process.

We can consider the woman who receives an adoptive baby or male couples who resort to temporary uterus surrogacy to have children: How do they achieve such a phenomenon that is essential to the baby at the beginning of life? How do mothers or parents emerge outside the uterine model?

We analyzed Winnicott’s conception and propose an adaptation to contemporary times: “whoever is available to receive the baby was once a baby who received care” - adding: the mother who gestated and is the genetic mother; the mother who did not gestate but is the genetic mother; the mother who gestated but is not the genetic mother; the adoptive mother; the adoptive father; the genetic father; the non-genetic father; the genetic father who gestated - “played mother and child or father and child, perhaps has younger siblings or cousins and helped care for them, or cared for babies of parents’ friends or younger brothers of friends at school. And perhaps has learned or read about childcare and may have firm opinions of his own about what is right and wrong in the treatment of babies” ([1969]1994, p. 199).

According to Winnicott (1994), mutuality, a concept referring to the beginning of communication between two people, is fundamental to the bond between mother (or caregiver) and baby. It is directly associated with healthy emotional development and human interactions, especially in the context of the caregiver-baby relationship. The baby is able to communicate with the caregiver because he is enabled by “inherited processes that lead him toward emotional development [...]” (p. 198).

Mutuality involves the interaction between a person capable of taking care of the absolute dependence of another being, the baby. “The ‘silent’ communication is of trustworthiness, protecting the baby from automatic reactions to intrusions of external reality” ([1969]1994, p. 201). Otherwise, such intrusions can cause trauma to the baby.

As the baby matures, the mother, father, or caregiver begins to fail gradually and tolerably. This process leads to differentiation between the self and others, allowing the baby to start experiencing mutuality as a more balanced interaction, where both can coexist.

## CONCLUSION

Gestation does not belong to femininity, but rather to individuals with a uterus. The biological capacity to become pregnant depends on the presence of a uterus; however, the meaning of this experience-as a personal lived reality-pertains to the subject’s intimate truth.

M.’s experience of pregnancy is inscribed in his subjective experience as a constitutive part of his masculinity, demonstrating that gender identifications are not restricted to bodily binarism but traverse the unconscious and the unique ways each subject inhabits their body.

M. did not experience an identity crisis due to the pregnancy, although complex psychic processes were set in motion. He paused hormone therapy in order to conceive, returned to a biologically female body, endured suffering, went through pregnancy, and had a vaginal birth. He allowed the emergence of anguish and permitted himself to be affected by it.

For M., the idea of having a genetic child was linked to a sense of existential continuity-a form of permanence beyond his own finitude. At the same time, this conception seems to sustain the belief that biological ties alone might be sufficient to generate affection within intersubjective relationships.

Studies on transgender people’s lived experiences in general, particularly in the context of pregnancy, are significant because they focus on individuals with their desires. As Lanz (2017) states, it is about “trans-being.”

The transgender man who gestates, the father who carries, is certainly a contemporary phenomenon that can provoke vertigo; however, we found that it is possible for a transgender man to gestate and care for his child and provide a safe, welcoming, and appropriate environment.

M.’s child’s life story may differ from that of most children, at least currently, but it is neither better nor worse. Currently, at two years of age, his child has received all the necessary care required to foster healthy emotional development.
